# Potential Utilization of a Polysaccharide from the Marine Algae *Gayralia oxysperma*, as an Antivenom for Viperidae Snakebites

**DOI:** 10.3390/md16110412

**Published:** 2018-10-27

**Authors:** Ana Cláudia Rodrigues da Silva, Maria Eugenia Rabello Duarte, Miguel Daniel Noseda, Luciana Garcia Ferreira, Juliana Emanuela Fogari Cassolato, Eladio Flores Sanchez, Andre Lopes Fuly

**Affiliations:** 1 Department of Molecular and Cellular Biology, Federal Fluminense University, Niterói, Rio de Janeiro 24020-141, Brazil; anacrs1@yahoo.com.br; 2 Department of Biochemistry and Molecular Biology, Federal University of Paraná, Curitiba, Paraná 81531-980, Brazil; mdn@ufpr.br (M.D.N.); lugarciaferreira@gmail.com (L.G.F.); jcassolato@yahoo.com.br (J.E.F.C.); 3 Laboratory of Biochemistry of Proteins from Animal Venoms, Research and Development Center, Ezequiel Dias Foundation, Belo Horizonte, Minas Gerais 30510-010, Brazil; eladiooswaldo@gmail.com

**Keywords:** snake venom, *Bothrops jararaca*, *Lachesis muta*, green marine alga, *Gayralia oxysperma*, polysaccharide, neutralization, antivenom

## Abstract

Worldwide, snakebites have serious implications for human health. The administration of antivenom is the official treatment used to reverse the toxic activities of envenomation. However, this therapy is not efficient to treat the local effects, leading to the amputation or deformity of affected limbs. As such, alternative treatments are needed. Here, we analyze the ability of a polysaccharide from the green marine alga *Gayralia oxysperma* (Go3) to inhibit the effects of venom from *Bothrops jararaca* and *Lachesis muta*. *B. jararaca* or *L. muta* venoms were incubated together with sulfated heterorhamnans from Go3, and the in vitro (coagulation, proteolytic, and hemolytic) and in vivo (hemorrhagic, myotoxic, edematogenic, and lethal) activities of venoms were assessed. Additionally, Go3 was injected before and after the injection of venoms, and the toxic activities were further tested. When incubated with the venoms, Go3 inhibited all activities, though results varied with different potencies. Moreover, Go3 neutralized hemorrhagic, myotoxic, and edematogenic activities when injected before or after injection with *B. jararaca* and *L. muta* venom. Go3 also blocked the coagulation of plasma in mice caused by the venoms in an ex vivo test. Therefore, Go3 has the potential to be used as antivenom for *B. jararaca* and *L. muta* bites, notably exhibiting higher efficacy on *L. muta* venom.

## 1. Introduction

Snakebites represent a global public health problem. According to the World Health Organization (WHO), snakebites are a neglected tropical disease that affects 5.5 million people annually, resulting in approximately 400,000 amputations and 120,000 deaths. However, epidemiological studies are inadequate and unreliable, resulting in such figures being underestimated [1,2,3]. In Brazil, most snakebites are caused by the family Viperidae (vipers), which includes the genera *Crotalus*, *Bothrops*, and *Lachesis*. The latter two genera are responsible for the highest incidence (90%) and lethality (1%), respectively [4]. Snake venoms are composed of a mixture of active and non-active enzymes that produce toxic and harmful local (pain, inflammation, tissue necrosis, and hemorrhage) and systemic effects (hemorrhage, blood coagulation disorders, renal, cardiac or pulmonary failures, and death) in victims [4]. As a consequence of these effects, victims may die or survive with permanent physical damage. Thus, the economic impact of snakebites on communities can be considerable, mainly because victims are, in general, the most economically productive [5].

To date, apart from supportive care, the official and effective treatment for envenomation by snakebite is the intravenous administration of antivenom (immunoglobulins) [6]. In most registered cases, antivenom efficiently neutralizes the systemic effects of venoms, preventing death. However, some factors may hinder the complete success of antivenom in inhibiting venom toxicity, such as delays in administration, inappropriate dosages, side effects (mild fever to anaphylactic reactions), and in rare situations, the incorrect choice of antivenom [7,8]. Moreover, some antivenom poorly neutralizes the local effects of venom, which may lead to deformities or amputations of affected limbs [9]. Therefore, the search for alternative or complementary treatments capable of minimizing or counteracting the toxic effects of snakebites without side effects is relevant, and deserves attention. In fact, there is a need to develop more effective antivenom and therapeutics to snakebites. Research of small molecule inhibitors and peptides, as well as recombinant antivenoms, has increased [10,11,12]. Natural products, primarily from plants, have been extensively investigated as antivenoms, and some have been used regularly as traditional medicine by indigenous communities for such purposes [13]. Although the marine environment has more living organisms than the terrestrial, it remains poorly investigated regarding the discovery of biologically-active molecules with antivenom effects. Among such organisms, seaweeds should be considered, since they produce a great diversity of molecules with pharmacological and biological effects, some of which are of medical and industry interest [14].

Among marine organisms, green algae have been extensively investigated due to their bioactive compounds, widespread distribution, and large biomass production. Sulfated polysaccharides obtained from the genera *Monostroma*, *Ulva*, and *Enteromorpha* are known to display a variety of biological activities, such as antiviral [15,16,17,18,19], antioxidant [20], antihyperlipidemic [21,22], and plant resistance-inducers [23]. Monostromatic marine algae belonging to the genus *Gayralia* (Chlorophyta) comprises two species: *G. oxysperma* and *G. brasiliensis* [24]. Some species related to the *Gayralia* genus, such as the monostromatic *Monostroma s*p., are cultivated and used in the food and cosmetics industries [25]. These species synthesize sulfated rhamnans that are principally constituted of 2-, 3-, or 4-linked rhamnosyl units. The sulfate groups are principally positioned on C-2, C-3, and C-4, or on both C-3 and C-4 [23,24,25,26]. Crude extract obtained from the green seaweed *Gayralia oxysperma* is composed of sulfated heterorhamnans [26]. *G. oxysperma* (Go3) was obtained by aqueous extraction at 80 °C (13.8 wt % yield, based on dried and milled seaweed), and presented a composition of 49.6% total carbohydrates and 17.0% uronic acids. Go3 is highly sulfated (25.3%), and presents rhamnose as the major monosaccharide, as well as minor amounts of xylose, glucose, galactose, glucuronic acid, galacturonic acid, and very low percentages of arabinose and mannose (59.0, 9.0, 10, 6, 11, 3, 1, and 1 mol %, respectively). Chemical and spectroscopic analyses performed with the major sulfated heterorhamnan constituent of Go3 (70%) demonstrated that 3-linked units are sulfated on C-2, C-4, disulfated, and unsulfated in a molar ratio of 1:077:038:0.46, respectively. The 2-linked rhamnosyl units are principally sulfated on C-4, sulfated on both C-3 and C-4, and unsulfated (1:0.37:0.50, respectively). Furthermore, glucuronic 2-sulfate and galacturonic acids and xylosyl units are components of the side chains of this partially-branched heterorhamnan. The heterorhamnans isolated from *G. oxysperma* exhibited potent antiviral activity against the herpes simplex virus (HSV-1 and HSV-2), and were devoid of cytotoxic effects at concentrations up to 1000 µg mL^−1^ when assayed on Vero cells [26]. Additionally, the range of biological activity of the heterorhamnans from *G. oxysperma* and their products (obtained by partial depolymerization) increased as they became cytotoxic against tumoral cells (U87MG). The inhibitory effect on human glioblastoma cells was correlated with the molecular weight and sulfate location of the partially depolymerized products [27]. Indeed, other polysaccharides of seaweed have been tested as antivenom; for example, a sulfated galactan and agaran from *Palisada flagellifera* and *Laurencia aldingensis*, respectively, were able to inhibit the toxic effects of *Lachesis muta* venom [28,29]. A fucoidan of the brown seaweed *Fucus vesiculosus* inhibited the myotoxic activity of some crotaline snake venoms; in these cases, the formation of a complex between fucoidan and isolated myotoxins has been postulated as the mechanism of action for this natural polysaccharide [30]. Surprisingly, smaller fucoidan molecules were not more efficient than larger ones at preventing muscle necrosis [31].

Therefore, we evaluated the effect of *G. oxysperma* (Go3) sulfated heterorhamnans against some toxic activities of *L. muta* and *B. jararaca* venoms.

## 2. Results

### 2.1. Inhibition of Go3 on In Vitro Assays of B. jararaca or L. muta Venoms

**Figure 1:**
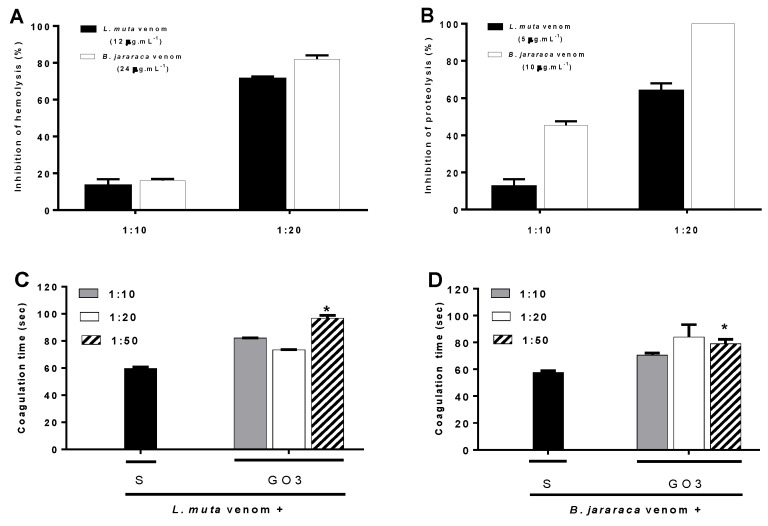
Inhibition by Go3 on in vitro assays of *L. muta* or *B. jararaca* venoms. *L. muta* and *B. jararaca* venoms were separately incubated with Go3 (1:10 and 1:20 venom:Go3 ratio (*w*/*w*), respectively), and then hemolysis (**A**) or proteolysis (**B**) assays were performed. 12 μg mL^−1^ of *L. muta* (**C**) and 24 μg mL^−1^ of *B. jararaca* (**D**) venoms (MCD of venoms) were incubated with saline (S) or Go3 (1:10, 1:20 or 1:50, venom:Go3 ratio (*w*/*w*)). Next, mixtures were added to plasma, and coagulation time was monitored. Data are expressed as means ± S.E. of three individual experiments (n = 3). * *p* < 0.05 when compared to control (black columns).

*L. muta* or *B. jararaca* (2–40 μg mL^−1^) venom induced hemolysis, proteolysis, or coagulation in a concentration-dependent manner. One minimum indirect hemolytic concentration (MIHC; 12 μg mL^−1^ for *L. muta* and 24 μg mL^−1^ for *B. jararaca*), effective concentration (EC; 5 μg mL^−1^ for *L. muta* and 10 μg mL^−1^ for *B. jararaca*), or minimum coagulation dose (MCD; 12 μg mL^−1^ for *L. muta* and 24 μg mL^−1^ for *B. jararaca*) of venoms was incubated with Go3 (as described in Materials and Methods), and then assays were performed. As shown in Figure 1A, regardless of the venoms tested, the inhibitory profiles of hemolysis by Go3 were similar; around 15% and 75% at 1:10 and 1:20 venom:Go3 ratios (*w*/*w*), respectively. Go3 inhibited *B. jararaca* proteolysis more efficiently than that of *L. muta*, since 100% of *B. jararaca* proteolysis was inhibited at 1:20 venom:Go3 ratio, whereas for *L. muta* venom, a 60% inhibition was achieved (Figure 1B). In contrast, the inhibition of coagulation by both venoms by Go3 was less effective, since a delay in coagulation (90 s, control value of 60 s) only occurred at the highest venom:Go3 ratio, which was 1:50 (Figure 1C). Go3 alone (at concentrations of up to 600 μg mL^−1^) did not cause hemolysis, proteolysis, or coagulation (data not shown).

### 2.2. Inhibition by Go3 of Ex Vivo Plasma Coagulation of L. muta Venom

The effect of Go3 on plasma coagulation of *L. muta* venom was evaluated using an ex vivo methodology (Table 1). Mice received a s.c. injection of Go3, saline, or *L. muta* venom. Two hours later, blood was collected and centrifuged, after which prothrombin time (PT) and activated partial thromboplastin time (aPTT) coagulation tests were performed. Moreover, another experimental approach was evaluated in which *L. muta* venom was injected s.c., and then followed 30 min later by an i.v. injection of Go3. After 90 min, blood was collected and coagulation tests were performed. As shown in Table 1, plasma did not clot if mice solely received an injection of *L. muta* venom, while the treatment of mice with Go3 restored plasma coagulation to normal levels in PT and aPTT tests. Injection of only Go3 did not alter plasma coagulation compared to mice that received a saline injection only.

### 2.3. Neutralization of Hemorrhage and Edema Caused by B. jararaca or L. muta Venoms

**Figure 2:**
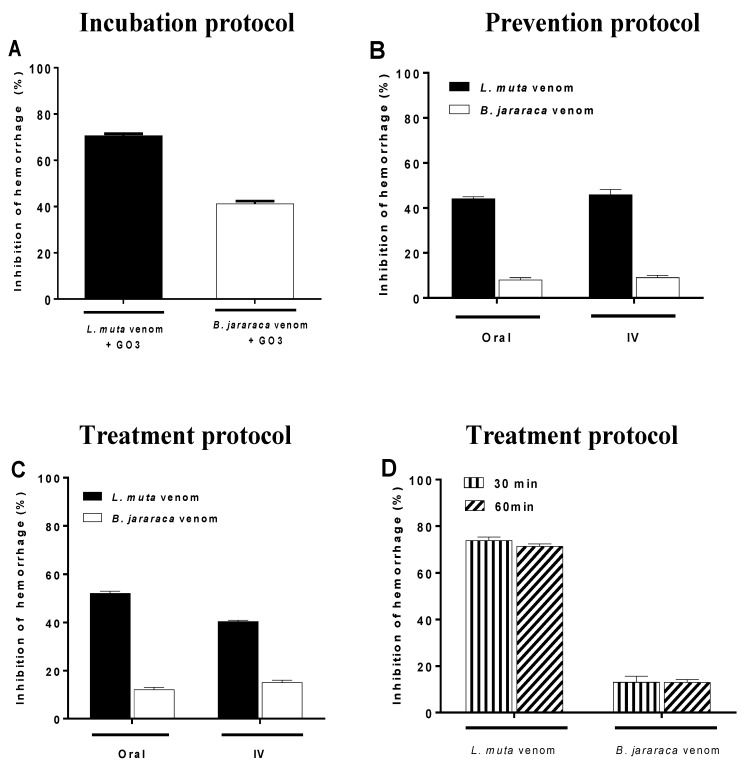
The inhibitory effect of Go3 on hemorrhage caused by *L. muta* or *B. jararaca* venoms. (**A**) Go3 (140 µg/mouse) was incubated with *L. muta* (24 µg/mouse) or *B. jararaca* (30 µg/mouse) venom for 30 min, and the mixture was then injected s.c. into mice; (**B**) Go3 was given orally or intravenously (i.v.), and mice received a s.c. injection of *L. muta* or *B. jararaca* venom 15 min later; (**C**) *L. muta* or *B. jararaca* venom was given s.c., and Go3 was administered orally or i.v. 15 min later; (**D**) *L. muta* or *B. jararaca* venom was injected s.c., and Go3 was injected s.c. 30 or 60 min later. Data are expressed as the mean ± SE of individual experiments (n = 5).

Subcutaneous injection of *L. muta* (24 µg/mouse) or *B. jararaca* (30 µg/mouse) venom induced a hemorrhage halo of 20 mm, which corresponds to two minimum hemorrhagic doses (MHDs), and such halos were considered to be 100% of hemorrhagic activity. When mixed with the venoms, Go3 (140 µg/mouse) inhibited 70% and 40% of the hemorrhagic activity of *L. muta* and *B. jararaca* venoms, respectively (Figure 2A). However, if *L. muta* (black columns) or *B. jararaca* (white columns) venoms were injected s.c. before (Figure 2B) or after (Figure 2C) the injection of Go3 (orally or i.v.), hemorrhage inhibition also occurred. The inhibitory effect of Go3 was more effective if Go3 was injected exactly at the same site for either *L. muta* or *B. jararaca* venoms, even if the Go3 was injected 30 or 60 min after the injection of these venoms (Figure 2D). Thus, the inhibitory effect of Go3 on the hemorrhagic activity of *L. muta* or *B. jararaca* venoms was optimal if Go3 was injected s.c. rather than orally or via the i.v. route, which was likely due to being more proximal to the venom injection site.

**Figure 3:**
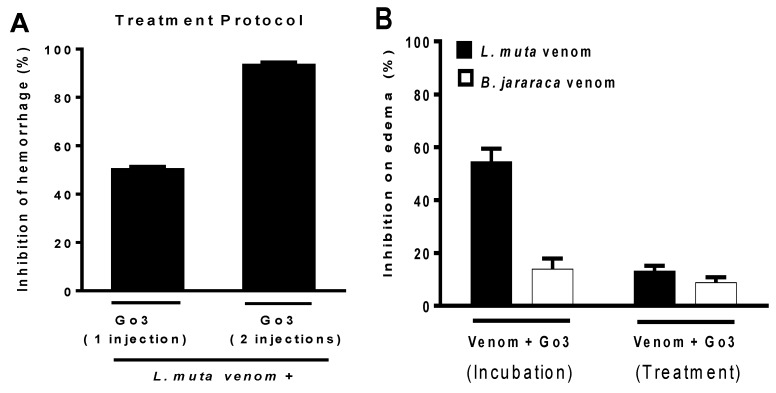
The inhibitory effect of Go3 on hemorrhage and edema caused by *L. muta and B. jararaca* venoms. (**A**) *L. muta* venom (24 µg/mouse) was given s.c. to mice, and then one or two injections i.v. of Go3 (140 µg/mouse) were performed (treatment protocol) 1 h later. 6 h after *L. muta* venom injection, hemorrhagic activity was measured using the aforementioned procedure; (**B**) At the incubation protocol, 13 µg/mouse of *L. muta* or *B. jararaca* venoms were incubated with 33 µg/mouse Go3, and mixture was then injected into mice. As part of the treatment protocol, *L. muta* (black column) or *B. jararaca* (white column) venom was injected s.c. into mice, and then mice received injection i.v. of Go3 15 min later. Edema was analyzed thereafter using the aforementioned procedure. Data are expressed as the mean ± SE of individual experiments (n = 5).

Since the neutralization of hemorrhagic activity by Go3 was more efficient with *L. muta* than *B. jararaca* venom, treatment protocols were only performed for the *L. muta* venom. *L. muta* venom (24 µg/mouse) was injected s.c., and a single injection (1 injection) or two injections (2 injections) of Go3 were given 1 h later (Figure 3A, treatment protocol). Then, after 6 h of *L. muta* venom injection, hemorrhage was analyzed using the method described above. As seen in Figure 3A, a single injection of Go3 inhibited 50% of *L. muta* -induced hemorrhage, while two injections of Go3 resulted in an inhibition of 90%. For edema, the injection of 13 µg/mouse of *L. muta* or *B. jararaca* venom into the paw of mice produced an increase of ca. 50% in paw volume, which was considered as being 100% of edematogenic activity. Subsequently, a similar dose of *L. muta* or *B. jararaca* venom was incubated with Go3 (33 µg/mouse) and then injected s.c. into mice, and then the edema was analyzed using the aforementioned procedure (Figure 3B). As shown in Figure 3B (incubation), Go3 inhibited edema caused by *L. muta* and *B. jararaca* venoms by ca. 55% and 15%, respectively. When Go3 was given after the injection of venoms, inhibitory values dropped to approximately 10% (Figure 3B, treatment). Neither injection of Go3 nor saline induced edema (data not shown).

### 2.4. Neutralization of B. jararaca Venom Lethality by Go3

Despite injecting large amounts of venom into victims, the lethal activity of *L. muta* venom is relatively weak. Thus, lethality was only tested with the venom of *B. jararaca*. Mice that received a single injection i.p. of *B. jararaca* venom (14 μg/mouse) mixed with saline died at 110 min (Table 2). In contrast, when *B. jararaca* venom was incubated with Go3 (140 μg/mouse) followed by i.p. injection of the mixture into mice, deaths occurred at 350 min. Go3 also delayed mice from death even when the Go3 injection was performed 15 or 30 min before (prevention protocol) or after (treatment protocol) injection of *B. jararaca* venom, and this neutralization efficacy was quite similar in both protocols (Table 2). Injection of only saline or Go3 (concentrations up to 500 μg/mouse) did not kill the mice (data not shown).

### 2.5. Neutralization L. muta Venom Myotoxicity by Go3

**Figure 4:**
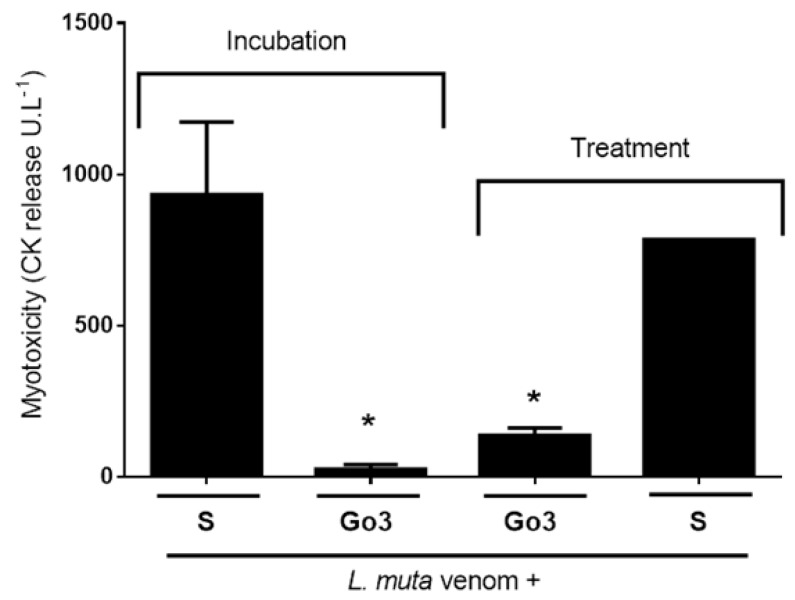
The effect of Go3 on myotoxicity caused by *L. muta* venom. For the incubation protocol, *L. muta* venom (15 μg/mouse) was incubated with saline (S) or with Go3 (140 μg/mouse), the mixtures were then injected into mice, and myotoxicity was analyzed by measuring CK release from mice plasma (expressed as U L^−1^). Alternatively (treatment protocol), *L. muta* venom was injected i.m., and Go3 or saline (S) was injected i.m. at the same site of the venom injection 15 min later. CK release was then measured as described. Data are expressed as the mean ± SE of individual experiments (n = 5). * *p* < 0.05 when compared to *L. muta* + saline (S).

Myotoxic activity was not performed for *B. jararaca* venom due to its low myotoxicity. As previously described, muscular tissue necrosis in victims caused by snakebite is directly associated with groups of enzymes of snake venoms, as low molecular weight basic toxins, phospholipase A_2_, hemorrhagic myotoxins, and cardiotoxins. The myotoxic activity of *L. muta* venom was analyzed through the release of creatine kinase (CK) from mice plasma. Injection i.m. of *L. muta* venom (15 μg/mouse) mixed with saline (S) produced a release of CK of the muscle of mice at of 950 U L^−1^ (Figure 4, incubation). When *L. muta* venom was mixed with Go3 (140 μg/mouse), the myotoxic activity of the venom was fully inhibited, since no trace of CK was detected (Figure 4, incubation). In another set of experiments (treatment), *L. muta* venom was injected i.m., and Go3 or saline (S) was injected 15 min later at the same site of the venom injection, with myotoxicity being evaluated thereafter. As seen in Figure 4, Go3 continued to protect mice from *L. muta* venom-induced myotoxicity through the treatment protocol. The injection of Go3 or saline only did not induce the release of CK of plasma in mice.

## 3. Discussion

The incidence and mortality indices of snakebites are higher than other neglected diseases, such as dengue, cholera, Chagas disease, leishmaniasis, yellow fever, and schistosomiasis [1,2]. Due to the unsatisfactory efficacy of classical antivenoms to counteract the local effects of venoms such as massive tissue necrosis [32,33], alternative treatments should be explored further, with natural organisms providing a good source of innovative bioactive molecules [13,14,34,35]. In general, the official protocol performed to test antivenoms involves incubating them with venoms and then injecting the mixture into mice to determine lethality. Undoubtedly, such procedures do not reflect a real situation of envenomation through snakebite, in which the venom is injected into victims and antivenom is administered afterwards. In fact, such a protocol may facilitate the inhibitory efficacy of antivenoms.

The present work demonstrated, for the first time, a sulfated heterorhamnans from a marine alga (Go3) being able to neutralize some of the in vivo activities (hemorrhage, edema, lethality, and myotoxicity) induced by *B. jararaca* and *L. muta* venoms. This is in contrast to both Brazilian antivenoms, which do not efficiently block tissue necrosis and other local effects of *B. jararaca* or *B. jararacussu* venoms (e.g., hemorrhage and inflammation) at clinical doses [36]. However, the inhibition of such in vivo activities was achieved only at higher concentrations of antivenom—nearly 10 times the dose used in cases of envenomation by *Bothrops* in Brazil [36,37]. With this in mind, pharmacokinetic and pharmacodynamic approaches should be performed to discover how Go3 acts in organisms, as well as the Go3 dosage necessary to treat victims. Understanding the observed neutralizing effect of Go3 remains an important task in the field of toxinology, since venom-induced edema, hemorrhage, and myotoxicity may lead to amputation and deformity; these effects are the main hazardous symptoms following snakebites and contribute to the progression of tissue damage. Go3 may prevent tissue necrosis induced by snakebite. The most notable result of this work relies on one chemical structure neutralizing different enzymes or active components of both venoms, and the inhibition of Go3 also occurring through two experimental protocols; the first was developed to more closely simulate a real envenomation (treatment), while the second protocol (prevention) was performed to provide prior protection to victims from the toxic effects of envenomation. In addition, regardless of the Go3 administration route, in vivo activities were also inhibited. The inhibition of toxic effects caused by the venom of the *L. muta* snake by other sulfated polysaccharides obtained from seaweeds was previously reported for sulfated galactans, and was correlated with possible interactions due to the negative and positive charges present in sulfated galactans and in the proteins present in snake venoms, respectively [28,29]. Additionally, a sulfated fucoidan from *Fucus vesiculosus* exhibited a protective effect against the cytotoxic and myotoxic effects of myotoxins from crotaline snake venoms [30,31], with a reduction in the molecular weight of fucoidan being accompanied by a reduction in antivenom activity. Therefore, higher molecular weights of such polysaccharides resulted in more efficient inhibition [31].

In particular, the inhibitory mechanism of action of Go3 should be investigated through binding to divalent metals such as Ca^2+^ or Zn^2+^. These metals should be considered because of their anionic charges, as well as their molecular weights. Most of the active enzymes in venoms require divalent metals to induce their toxic activities [37]. However, regardless of the inhibition mechanism, Go3 may be effective against other species of *Bothrops* or *Crotalus*, as their venom composition is quite similar. The structural features (molecular weight, glycoside linkage, sugar composition, and the content of sulfate groups) are critical for polysaccharides to display pharmacological activities [19,22,38,39]. This is true for polysaccharides isolated from green seaweeds, in which the presence of 2-linked rhamnosyl units that are 3,4-sulfated allows the binding to target on plasma membrane or intracellular proteins of tissues [24]. The literature also indicated that sulfated polysaccharides from seaweeds have little to no cytotoxicity to cells [40], and could thus be good antivenom candidates.

## 4. Materials and Methods

### 4.1. Snake Venom, Animals, and Other Materials

The venoms of *L. muta* and *B. jararaca* were kindly supplied by Fundação Ezequiel Dias (FUNED), Belo Horizonte, Minas Gerais State, Brazil, and stored at −20 °C until use. Balb/c mice (18–20 g) were obtained from the Núcleo de Animais de Laboratório (NAL) of the Federal Fluminense University (UFF), and were housed under constant temperature (24 ± 1 °C) and light conditions. Experiments were approved by the UFF Institutional Committee for Ethics in Animal Experimentation (protocol number 25), which are in accordance with the guidelines of the Brazilian Committee for Animal Experimentation (COBEA). All reagents were of the best available grade.

### 4.2. Collection of G. oxysperma Specimens

Specimens of *G. oxysperma* (Kützing) K. L. Vinogradova ex Scagem et al. were collected on the southern coast of Brazil (Bahia de Paranaguá, Paraná State). An exemplar specimen was deposited at the herbarium of the Federal University of Paraná (Curitiba, Brazil) under the herbarium identification code UPCB-58059.

### 4.3. Extraction and Purification of Go3 Sulfated Heterorhamnans Fraction

The algal material was cleaned, washed with tap water, sun-dried, and milled. The polysaccharides were extracted as previously described [26,27]. Briefly, dried and milled *G. oxysperma* was extracted twice with water (5%, *w*/*v*) at 25 °C for 4 h under mechanical stirring. After centrifugation, the supernatant was treated with ethanol (EtOH) (3:1, *v*/*v*), and the EtOH- precipitated material was redissolved in distilled water, dialyzed (cut-off at 12–14 kDa), concentrated by rotary evaporation under reduced pressure, and freeze-dried, giving rise to the polysaccharide extract named “Go1” (1.4% yield, based on dried and milled seaweed). This entire process was repeated to create Go2 extract (0.6% yield). The algal residue obtained from extractions at 25 °C was then submitted to similar aqueous extraction at 80 °C, giving rise to the Go3 fraction (13.8% yield). The sulfated heterorhamnans Go3 (10 mg) was resuspended in 1 mL of saline, aliquoted, and stored at −20 °C until assays were performed.

### 4.4. In Vitro Assays

#### 4.4.1. Antihemolytic Activity

The degree of hemolysis caused by *L. muta* or *B. jararaca* venom was determined by indirect hemolytic test using human erythrocytes and hen’s egg yolk emulsion as substrate that contains phospholipids [41]. After incubating venoms with substrate (phospholipids) for 15 min at 37 °C, the reaction was stopped by adding EDTA (8 mM, final concentration). Then, a washed red blood cells suspension (2 % *v*/*v*) was added to the reaction medium and tubes were incubated for 1 h at 37 °C. At this moment, lysolecithin formed during the enzymatic reaction lyzed cells, tubes were centrifuged at 2000 rpm for 10 min, and released hemoglobin was measured at Absorbance 578 nm. After creating a concentration-response curve, the amount of venom (μg mL^−1^) capable of inducing 100% of hemolysis was denoted as the minimum inhibitory hemolytic concentration (MIHC). Then, one MIHC of *L. muta* (12 μg mL^−1^) or *B. jararaca* (24 μg mL^−1^) venom was incubated with Go3 (at 1:10 or 1:20 *w*/*w*, venom:Go3) for 30 min. at 25 °C, followed by the hemolytic test. Control experiments were performed by incubating venoms with saline in the absence of Go3, or by adding Go3 alone to reaction medium.

#### 4.4.2. Antiproteolytic Activity

The proteolytic activity of *L. muta* or *B. jararaca* venom was determined as in [42], using azocasein (obtained from Sigma Chemical Co., St. Louis, MO, USA) as a substrate (0.2% *w*/*v* in 20 mM Tris-HCl, 8 mM CaCl_2_, pH 8.8) with modifications. Different venom concentrations were incubated with 0.4 mL azocasein at 37 °C for 90 min in a total volume of 1.2 mL. The enzymatic reaction was stopped by the addition of trichloracetic acid (5% *v*/*v*, final concentration). The tubes were centrifuged at 15,000× *g* for 3 min. The supernatant was then removed and mixed with 2 M NaOH, and the tubes were read at Absorbance (A) 420 nm to measure the release of azo dye. The effective concentration (EC) was defined as the amount of venom (μg mL^−1^) able to produce a variation of 0.2 units at A 420 nm. For the inhibitory experiments, one EC of each venom (5 μg mL^−1^ for *L. muta* and 10 μg mL^−1^ for *B. jararaca*) was incubated with Go3. Proteolytic activity was then determined accordingly.

#### 4.4.3. Anticoagulant Activity

Different concentrations of *B. jararaca* or *L. muta* venom were added to plasma, and coagulation time was monitored using a digital Amelung coagulometer (model KC4A, Labcon, Germany). The amount of venom (μg mL^−1^) able to clot plasma around 60 s was called minimum coagulation dose (MCD), and venom (*L. muta*, 12 μg mL^−1^ or *B. jararaca*, 24 μg mL^−1^) was incubated with different concentrations of Go3, resulting in venom:Go3 ratios (*w*/*w*) of 1:10, 1:20 or 1:50 for 30 min at 25 °C. Following incubation, this mixture was added to the reaction medium and coagulation was monitored as previously described above. Controls were performed by adding Go3 or saline with plasma in the absence of venom.

#### 4.4.4. Ex Vivo Coagulation Tests

Three different experimental groups were performed, as follows: (a) *L. muta* (24 µg/mouse) venom was administered subcutaneously (s.c.) into the abdomen cavity of mice; (b) Go3 (140 µg/mouse) or saline was administered intravenously (i.v.); or (c) *L. muta* venom was administered s.c., and Go3 was injected i.v. 30 min later. Regardless of the experimental group, animals were euthanized after 2 h and blood was collected by cardiac puncture using citrate as anticoagulant. Blood was centrifuged at 1800 *g* for 10 min, and plasma was transferred to plastic tubes. Then, prothrombin time (PT) or activated partial thromboplastin time (aPTT) tests were performed according to the manufacturer’s instructions (Wiener Laboratories, Rosario, Argentina). For PT, an aliquot (50 μL) of plasma was incubated for 1 min at 37 °C, and then 100 μL of pre-warmed thromboplastin with calcium were added to initiate coagulation. For the aPTT test, plasma was incubated for 1 min at 37 °C with 100 μL of the aPTT reagent and cephalin plus kaolin for a final volume of 200 μL. Coagulation was then triggered by adding CaCl_2_ (8.3 mM, final concentration) and monitored on the coagulometer.

### 4.5. In Vivo Assays

#### 4.5.1. Antihemorrhagic Activity

Hemorrhagic lesions produced by *L. muta* or *B. jararaca* venom were quantified using a procedure described by [43], with some modifications. *B. jararaca* or *L. muta* venom was injected intradermally (i.d.) into the abdominal skin of mice, and animals were euthanized by decapitation 2 h later. The abdominal skin was removed, stretched, and inspected for visual changes in the internal aspect in order to localize hemorrhagic areas. One minimum hemorrhagic dose (MHD) was defined as the amount of venom (µg/mouse) able to produce a hemorrhagic halo of 10 mm, which was 12 and 15 µg/mouse for *L. muta* and *B. jararaca* venom, respectively. To analyze the effect of Go3 on venom-induced hemorrhaging, three protocols were employed: (a) incubation: Go3 was mixed with *L. muta* (24 µg/mouse) or *B. jararaca* (30 µg/mouse) venom for 30 min at 25 °C, and the mixture was then injected i.d. into mice; (b) prevention: Go3 was administered orally or i.v., and venom was injected s.c. into mice 15 min later; (c) treatment, venom was injected s.c., and Go3 was administered orally or i.v. 15 min later. Additionally, venom was injected s.c., and Go3 was injected s.c. at the site of venom injection 30 or 60 min later.

Moreover, two additional sets of experiments were performed: (a) *L. muta* venom was injected s.c., and Go3 was administered i.v. 1 h later. After 6 h, animals were euthanized and hemorrhagic activity was assessed; (b) *L. muta* venom was injected s.c., and Go3 was injected i.v. 1 or 2 h later. After 6 h, animals were euthanized and hemorrhagic activity was assessed. Negative controls were performed by injecting solely saline or Go3 instead of venom. The volume of sample injections into mice was 100 µL.

#### 4.5.2. Antiedematogenic Activity

The edema-inducing activity of venom was determined according to [44] with some modifications. Groups of five mice received a single s.c. sub plantar injection of 50 μL of *L. muta* or *B. jararaca* venom into the right paw, while the left paw received 50 μL of saline. Then, edema was evaluated 1 h after injection and expressed as the percentage increase in the weight of the right paw (ca. 50%) compared to the left one. The effect of Go3 was evaluated by mixing venoms with Go3 for 30 min at 25 °C, followed by the mixture (50 μL) being injected s.c. into animals. Additionally, the protocol for treatment was employed, in which *L. muta* or *B. jararaca* venom was injected s.c., and Go3 was injected i.v. 15 min later. Edema was then measured. The control group was injected with Go3 or saline in the absence of venom.

#### 4.5.3. Antilethality Activity

The antilethality assay was performed by incubating *B. jararaca* venom (14 μg/mouse) with Go3 (140 μg/mouse) for 30 min at 25 °C, followed by the mixture being injected i.p. into mice. Positive groups received *B. jararaca* venom mixed with saline, while the negative control received solely Go3 or saline instead of venom. Similarly, two other protocols were also performed: (a) treatment: *B. jararaca* venom was injected i.p., and Go3 was injected via the same route 15 or 30 min later; and (b) prevention: Go3 was injected i.p., and *B. jararaca* venom was injected i.p. 15 or 30 min later. After the end of injections, the number of deaths was observed over a period of 24 h. The injection volume of samples was 100 μL.

#### 4.5.4. Antimyotoxic Activity

*L. muta* venom or saline was injected (50 μL) intramuscularly (i.m.) into mice under the right tibial anterior muscles, so that the injected site was positioned just over the *extensor digitorum longus* (EDL) muscle [45]. After 2 h, blood was collected and the serum was separated by centrifugation and stored at 4 °C for subsequent determination of the plasma CK activity using a diagnostic kit (Sigma Chemical Co., Saint Louis, MO, USA). The rate of CK release from the isolated muscles was expressed as the increase in CK release compared to control values (mice that received a saline injection or did not receive any other injection). CK activity was expressed as international units (U), where 1 U is the enzyme amount that catalyzes the transformation of 1 μmol of substrate at 25 °C. The rate of CK release from the isolated muscle was expressed as enzyme units released into the medium per gram per h of collection (U/g h), as previously reported [45]. Myotoxicity was then expressed as the increase of released CK. The inhibitory effect of Go3 was assessed using the following protocols: (a) incubation: *L. muta* venom (15 μg mL^−1^) was incubated with Go3 (140 μg mL^−1^) or saline for 30 min at 25 °C, and the mixture (50 μL) was then injected i.m. into mice; (b) treatment: *L. muta* venom (50 μL) was injected i.m., and Go3 (50 μL) was injected i.m. at the venom injection site 15 min later. The negative control was performed by injecting saline instead of Go3, and myotoxicity was determined 2 h later, as described here.

### 4.6. Statistical Analysis

Results were expressed as the mean ± SE obtained from the number of animals or experiments performed. The statistical significance of differences among experimental groups was evaluated using the Student’s *t*-test. *p* values < 0.05 were considered significant.

## 5. Conclusions

Overall, Go3 appeared to inhibit the primary toxic activities of *B. jararaca* and *L. muta* venom. These results suggest that the use of Go3 as medicine or phytotherapy to improve antivenom therapy could prevent or diminish the effects of local necrosis that cause severe disabilities, disfigurement, or mortality among snakebite victims. In order to achieve this goal, the development of dosage forms (as cream, gel, capsule, patch or pill) using Go3 should be investigated further.

## Figures and Tables

**Table 1:** Effect of Go3 on the ex vivo coagulation of mouse plasma by *L. muta* venom.

Groups	Coagulation Test (sec)	
	aPTT (s)	PT (s)
Go3 + saline ^a^	44 ± 0.5 *	18 ± 0.4
*L. muta* + saline ^a^	800 ± 0.1 *	800 ± 0.1 *
*L. muta* + Go3 ^b^	45 ± 0.3 *	21 ± 0.4
Saline ^a^	27 ± 0.7	19 ± 0.6

^a^ Mouse groups received a s.c. injection of Go3 with saline, or *L. muta* venom (24 µg/mouse) with saline, or saline alone. ^b^ *L. muta* venom was injected s.c. into mice; 30 min later, Go3 was injected i.v. * Statistical significant difference (*p* < 0.05 when compared to saline alone). Results are expressed as the mean ± SE of two individual experiments (n = 4).

**Table 2:** The protective effect of Go3 on lethality produced by *B. jararaca* venom.

Groups	Survival Time for Different Protocols (min)				
	Incubation ^a^	Prevention ^b^		Treatment ^c^	
	30 min	15 min	30 min	15 min	30 min
*B. jararaca* venom + saline	110 ± 20	101± 15	101 ± 11	101 ± 17	101 ± 18
*B. jararaca* venom *+* Go3	350 ± 25 *	205 ± 27 *	215 ± 17 *	206 ± 21 *	189 ± 22 *

^a^* B. jararaca* venom (14 μg/mouse) was incubated with saline or with Go3 (140 μg/mouse) for 30 min at 25 °C, and then the mixture was injected i.p. into mice; ^b^ Go3 was injected i.p., and *B. jararaca* venom was injected 15 or 30 min later via the same route; ^c^ *B. jararaca* venom was injected i.p., and Go3 was injected 15 or 30 min later via the same route; ^b,c^ Results are expressed as the mean ± SE of two individual experiments (n = 6). * *p* < 0.05 when compared to *B. jararaca* + saline.
